# A Literature Review and Case Report of Metastatic Pure Choriocarcinoma

**DOI:** 10.1155/2015/345018

**Published:** 2015-11-16

**Authors:** Diwei Lin, Amanda Jia Hui Tan, Rajinder Singh-Rai

**Affiliations:** Division of Surgery, Lyell McEwin Hospital, Adelaide, SA, Australia

## Abstract

In 2012, testicular cancer was estimated to account for 940 disability adjusted life years in Australia; of these, 450 were years lost due to premature death and 500 were years of healthy life lost due to disease, disability, or injury (Australian Institute of Health and Welfare and Australasian Association of Cancer Registries, 2012). Testicular choriocarcinoma is one of the rarest variants of testicular germ cell tumours, accounting for less than 1% of testicular germ cell tumours and only about 0.19% of all testicular tumours. Management involves radical orchidectomy and chemotherapy. Even then, prognosis is poor. This case report describes a 20-year-old male with pure testicular choriocarcinoma with pulmonary metastases which showed sustained and complete response to adjuvant chemotherapy consisting of bleomycin, etoposide, and cisplatin.

## 1. Introduction

In 2012, testicular cancer was estimated to account for 940 disability adjusted life years in Australia; of these, 450 were years lost due to premature death and 500 were years of healthy life lost due to disease, disability, or injury [1]. Testicular cancer is the most common malignancy in men aged 15 to 34 years and accounts for approximately 1% of all cancer in men [2]. In 2009, there were 751 new cases of testicular cancer in Australia, accounting for 1.2% of all newly diagnosed cancer in men [1].

Over the last 4 decades, the observed incidence of testicular cancer has more than doubled. However, increasingly effective treatment has seen rates of direct mortality from testicular cancer drop significantly despite the rising incidence of testicular cancer [3]. It is estimated that 69% of testicular cancers are localised, 18% are regional, and 12% are found to be metastatic at the time of diagnosis [4]. Testicular cancer can be broadly divided into germ cell tumours (GCTs) and non-germ cell tumours (NGCTs). GCTs account for 94% of testicular tumours and include 5 basic cell types: seminoma, embryonal carcinoma, yolk sac tumour, teratoma, and choriocarcinoma. Of the 5 types, pure choriocarcinoma is regarded as the rarest [5]. Even though choriocarcinoma is not uncommonly seen as a component of a nonseminomatous germ cell tumours (NSGCTs), pure choriocarcinoma is very rarely identified [6]. This case report discusses a 20-year-old male with a rare finding of pure testicular choriocarcinoma with pulmonary metastases.

## 2. Case Presentation

A 20-year-old male was admitted by the urology team after being referred by his GP with a 6-month history of increasing right testicular swelling that had been gradually increasing in size. The patient had no significant past medical or surgical history of note and no family history of any cancer. This patient also had no history of cryptorchidism.

On further history, the patient described the swelling as initially being painless. However, over the last 1 month, he began developing a dragging discomfort in his right groin associated with a rapid increase in the size of the right testis. This was not associated with any systemic symptoms, weight loss, or lower urinary tract symptoms.

On examination, the right testis was found to be firm and solid with no palpable paratesticular lumps. The swelling was not separate from the testis, not transilluminable, and the cord was palpable on top of the swelling. A scrotal ultrasound performed showed that his right testis was grossly enlarged and heterogenous in echotexture, measuring 6.1 × 6 × 5.5 cm. There was also increased Doppler flow within the right testis reflective of likely primary testicular cancer.

Initial tumour markers showed a HCG of 163000 U/L with AFP of <2 kU/L. Strongyloides, hepatitis, and HIV serology were negative.

Staging CT scans revealed multiple lung nodules bilaterally consistent with metastases. The largest deposits were 14 mm and 13 mm in the left lower lobe and right upper lobe posteriorly, respectively. A total of between 10 and 15 metastatic deposits were noted in the lungs with no metastasis identified in the abdomen or in the pelvis. A CT scan of his head was also performed which showed no acute intracranial pathology, masses, or lesions. He proceeded to have a radical orchidectomy followed by a chemotherapy regime consisting of bleomycin, etoposide, and cisplatin.

**Figure 1:**
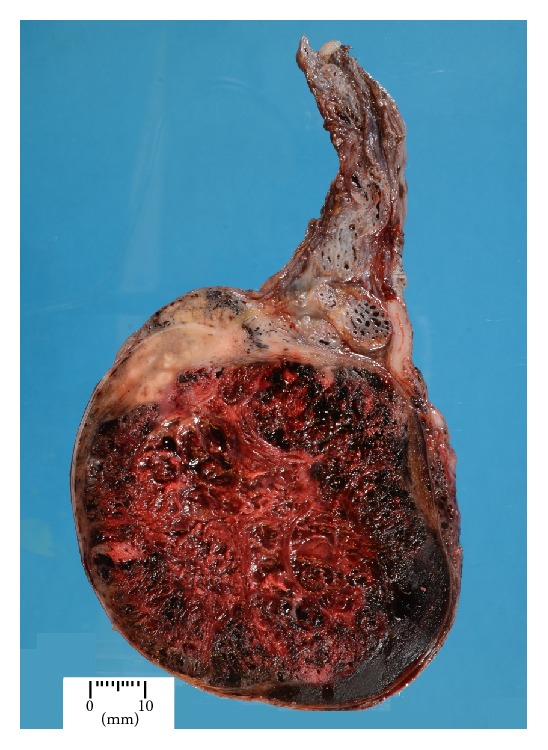
Gross image of the resected testis demonstrating replacement of the parenchyma by a diffusely haemorrhagic, centrally necrotic tumour.

**Figure 2:**
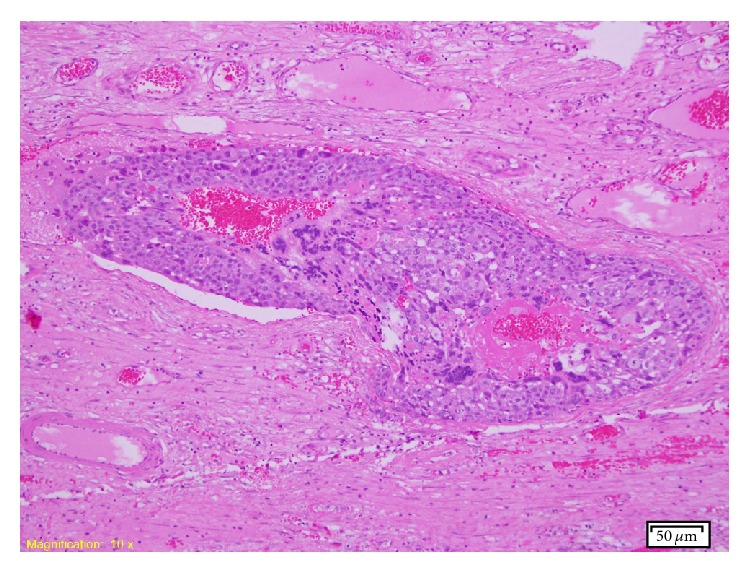
Entire tumour composed of choriocarcinoma characterised by an admixture of syncytiotrophoblastic and cytotrophoblastic cells.

Histological macroscopic examination showed an enlarged testis which was almost completely replaced by an extensively haemorrhagic, centrally necrotic tumour which measured 76 × 74 × 53 mm (Figure 1). The spermatic cord was normal in appearance. The testicular mass was extensively sampled and representative sections were taken from the spermatic cord. Microscopic examination revealed that the entire tumour was composed of choriocarcinoma characterised by an admixture of syncytiotrophoblastic and cytotrophoblastic cells (Figure 2). The syncytiotrophoblastic cells were positive for HCG and both components were negative for AFP, CD30, and cytokeratins. No other germ cell tumour elements were identified in the many sections examined. Vascular invasion was present in several areas and focal rete testis infiltration was present. There was no invasion of the tunica albuginea or the spermatic cord. Intratubular germ cell neoplasia (ITGCN) was also present in the remnant testicular parenchyma. The AJCC TNM system stage was pT2 N0 M1a.

Following initiation of chemotherapy and at 6 months after diagnosis, the patient continues to show sustained remission, with complete resolution of pulmonary metastatic disease and normalisation of tumour markers (HCG 8.6 U/L).

## 3. Discussion

The prognosis of metastatic pure testicular choriocarcinoma has traditionally been regarded as being extremely poor, with most authors describing dismal outcomes [7, 8]. We believe this case is significant as it is one of only a few published reports showing a positive outcome in a patient who presented with an advanced stage metastatic pure testicular choriocarcinoma.

Choriocarcinoma is a tumour of germ cell origin which recapitulates the developing trophoblast. About 8% of mixed germ cell tumours contain a component of choriocarcinoma [7]. Pure choriocarcinoma is extremely rare and represents less than 1% of testicular GCTs and only about 0.19% of all testicular tumours. In a literature review of 10,000 cases of GCTs, Ramón y Cajal et al. found just 54 (0.5%) cases of pure choriocarcinoma [8]. The estimated incidence of choriocarcinoma, occurring either in a pure form or as a component of a mixed germ cell tumour, is approximately 0.8 cases per year per 100,000 male population in countries with the highest frequency of testicular cancer [9].

Whilst choriocarcinoma has been noted to occur in men at any age after puberty, it is most often seen in young patients averaging 25–30 years of age [9]. Clinically, testicular choriocarcinomas present as a swelling in the testis associated with hormonal symptoms such as gynaecomastia and elevated serum human chorionic gonadotropin (HCG) levels, both of which were seen in this patient. Interestingly, choriocarcinomas have been found to be small swellings or even “burned-out,” with only a fibrous residual scar with minimal viable tumour at the time of orchidectomy. This is a result of spontaneous involution. This patient however had a large testicular swelling that had been increasing in size in the last 6 months.

Microscopically, choriocarcinoma is composed of atypical syncytiotrophoblastic and cytotrophoblastic cells which are intimately admixed. This is in contrast to other germ cell tumours of the testis, such as classical seminoma, in which scattered syncytiotrophoblast cells may be seen unaccompanied by cytotrophoblast. When stained immunohistochemically, the syncytiotrophoblastic cells of choriocarcinoma are positive for the HCG antigen. This is in keeping with the usual clinical finding of raised serum HCG. They are negative for AFP and for cytokeratins, OCT4, SOX-2, and CD117.

On gross examination, choriocarcinoma appears as an irregular lesion, often necrotic and usually diffusely haemorrhagic. Sampling of all testicular tumours is undertaken with a view to demonstrating whether the tumour invades structures such as the rete testis, epididymis, spermatic cord soft tissues, and/or the tunica albuginea. However, choriocarcinoma has a proclivity for vascular invasion which is in keeping with the embryological function of the normal trophoblast. It is this property which is thought to account for the tumour's proclivity to early, widespread haematogenous dissemination. For this reason although choriocarcinoma of the testis is staged in the same manner as all other testicular tumours, the presence or absence of vascular invasion is of most clinical relevance. Nodal metastases also occur but are less common.

Cryptorchidism has a well-established link and is regarded as the most significant risk factor for the development of testicular cancer, increasing risk by upwards of 10-fold [10]. In a case series comprising 125 patients with testicular cancer and a past history of cryptorchidism, 3 (2%) of the tumours were found to be pure choriocarcinomas, slightly higher than the overall incidence of choriocarcinomas among GCTs (about 1%). However, given the very few published cases of pure choriocarcinomas in the literature, it is not possible to hypothesise if cryptorchidism increases the likelihood of developing choriocarcinomas [11].

Radical orchidectomy and histological assessment remains the mainstay of therapy for testicular cancer, with CT scanning shown to be the most sensitive method for detecting metastatic disease in secondary sites. Positron emission tomography (PET) is an emerging diagnostic modality that could be used to assess for metastatic disease, but its optimal role has yet to be established [12].

Testicular choriocarcinomas have been found to be highly malignant and carry the worst prognosis of all GCTs [6]. They have the potential for early haematogenous metastases due to vascular invasion. Metastases occur most often to the lungs, with metastases to other organs such as the liver, gastrointestinal tract, and brain also described [13].

Tsuchiya et al. described the first case of pure testicular choriocarcinoma in 1980 [14]. Since then, the English literature has only contained case reports of pure testicular choriocarcinoma, with no published case series. Current management is based on anecdotal evidence and a thorough search of current literature has found no established guidelines regarding management of these patients. Cisplatin-based therapies are the mainstay of treatment for patients with metastatic NSGCTs, with quoted cure rates of up to 80% for advanced disease and nearly 100% for early-stage disease [15]. However, clinicians in case reports have found that pure choriocarcinomas are not as sensitive to chemotherapy as NSGCTs, possibly a reflection of its high malignant potential and poor prognosis associated with early haematogenous metastasis and its associated complications [16]. Nonetheless, standard chemotherapy regimens comprising bleomycin, etoposide, and cisplatin (BEP) for 4 cycles still remain the initial treatment for pure choriocarcinomas.

As discussed previously, prognosis of pure testicular choriocarcinoma is poor. Batata et al. in 1982 reported a 5-year survival rate of 0% in an analysis of 20 patients with choriocarcinoma [17]. In patients with brain metastases from testicular choriocarcinoma, a literature review by Vugrin et al. in 1979 found 5 such patients of whom all died [18]. The median survival among these 5 patients was just 1 month despite treatment with multiagent chemotherapy. Nonetheless, there have been sporadic case reports noting some success with treatment of testicular choriocarcinoma. Brigden et al. in 1982 described a case of a 19-year-old male with pure choriocarcinoma and lung metastases at time of diagnosis in which they managed to achieve complete response and sustained remission with a regime of cyclophosphamide, vinblastine, actinomycin D, bleomycin, and cisplatin [19]. More recently, Sharma et al. have described successful treatment of a similar patient using a BEP regime, with no evidence of recurrence 3 years after initial diagnosis of metastatic testicular choriocarcinoma.

In summary, this is a 20-year-old male who presented with an extremely rare case of metastatic testicular choriocarcinoma which responded to a regimen of BEP. His clinical presentation was interesting because of his complete response to treatment despite an advanced stage of disease at presentation. The authors hope a future case series could potentially describe long-term survival outcomes after BEP chemotherapy in pure testicular choriocarcinoma.
